# Between-Session Reliability of Portable Isometric Mid-Thigh Pull and Countermovement Jump Tests in Elite Male Ice Hockey Players from the Swedish Hockey League

**DOI:** 10.3390/sports13120456

**Published:** 2025-12-18

**Authors:** Manne Godhe, Sebastian Bergman, Henrik Petré

**Affiliations:** 1 Department of Physical Activity and Health, The Swedish School of Sport and Health Sciences, 114 33 Stockholm, Sweden; 2 Department of Physiology, Nutrition, and Biomechanics, The Swedish School of Sport and Health Sciences, 114 33 Stockholm, Sweden

**Keywords:** reproducibility, isometric peak force, fatigue, performance, injury prevention

## Abstract

This study investigated the test–retest reliability of strength and power-related measures assessed with a portable IMTP set-up and with CMJ in elite ice hockey players from the Swedish Hockey League. Twenty-two male ice hockey players (age: 26.8 ± 5.1 yr; height: 184.5 ± 3.9 cm; body mass: 88.6 ± 5.7 kg) participated in this study. The participants performed three maximal IMTP and CMJ trials on two separate occasions. Absolute and relative variables from the portable IMTP (force and rate of force development) and CMJ (force, power, velocity, impulse, jump height, time to peak force, time to peak power, concentric duration and eccentric duration) were obtained using force plates. Excellent reliability (ICC > 0.90; CV < 5%) was observed for multiple CMJ parameters, such as peak force (ICC = 0.94; CV = 2.7%), concentric and eccentric impulse (ICC = 0.96; CV = 1.4% resp. ICC = 0.95; CV = 2.9%) and CMJ peak power (ICC = 0.93; CV = 2.3%). IMTP peak force also demonstrated excellent reliability (ICC = 0.95; CV = 2.4%). IMTP rate of force development variables yielded reliability ranging from poor to moderate (CV = 12.9–54.6%). CMJ and portable IMTP provide highly reliable assessments of most strength and power-related variables in elite male ice hockey players. While absolute peak impulse, velocity and force, power and concentric duration displayed the highest reliability and should be prioritized, RFD variables require cautious interpretation due to high variability.

## 1. Introduction

Isometric mid-thigh pulls (IMTP) and countermovement jumps (CMJ) are widely utilized by practitioners and researchers to assess strength and power-related variables, offering valuable insights for training program adjustments, fatigue monitoring, kinetic data assessment, and return-to-play decision-making [1,2]. These measurements are often selected because they are informative, time-efficient, safe and easy to perform in different environments [3]. Force plates are considered the gold standard for both IMTP and CMJ assessments and are extensively used in sport science due to their ability to capture highly precise and informative data. They provide detailed measurements of both outcome-oriented variables, such as jump height, peak force, power, impulse, and velocity, which are strongly correlated with key performance indicators like linear speed and change in direction [4,5,6], as well as time-dependent variables, such as time to peak force, time to peak power, and the durations of concentric and eccentric phases, which offer deeper insights into neuromuscular function and are particularly effective for detecting fatigue-related changes [7,8]. Traditionally, IMTP assessments are conducted in laboratory settings using force plates and a fixed bar mounted to a squat rack, ensuring stability in both anterior–posterior and medial-lateral directions. However, portable IMTP setups, featuring a compact platform, adjustable chain, and lightweight pulldown bar, may provide a feasible alternative in field environments [9]. These portable systems offer a practical, space-efficient, and safe alternative for strength testing. When integrated with force plates, they enable seamless execution of both IMTP and CMJ assessments in the same location and in immediate succession, enhancing efficiency and data consistency.

To ensure that observed performance changes reflect genuine improvements rather than measurement error, it is essential to establish the reliability of the methods. This involves assessing test–retest consistency and defining sensitivity thresholds that can detect meaningful variations. Knowledge of the precision in the assessments is particularly vital in elite athletes since physiological responses to training are generally smaller in this population compared to less trained individuals [10]. Given that biological variability is considered the primary source of measurement variance [11], and force and force–time characteristics may differ across sports and between genders [12], it is essential to establish the reliability of these methods within specific athletic populations. This is especially critical in sports that are dependent on strength and power, where such measurements are commonly utilized.

Ice hockey places high demands on athletes, requiring repeated high-intensity sprint efforts interspersed with recovery periods [13]. To meet these demands, players must generate substantial force, a capacity reflected in studies showing strong physical profiles among ice hockey athletes [14]. Skating performance has been linked to both maximal strength [15,16] and vertical jump ability [17,18,19]. In practice, these qualities are commonly assessed outside laboratory settings using portable IMTP and CMJ tests. Consequently, establishing the reliability of these methods in ice hockey players, especially the inter-session reliability, is essential for accurate performance evaluation and informed training decisions. Without knowledge of the inter-session reliability of these measurements, coaches risk misinterpreting data, leading to poor training adjustments or missed injury signals.

Strength- and power-related variables collected with a fixed conventional isometric mid-thigh pull set-up [3,20] and CMJ [3,21] have been investigated and considered reliable in athletes across various sports [3,21]. However, most studies have focused on general athletic populations rather than sport-specific elite cohorts. Given that ice hockey players are likely to develop unique neuromuscular profiles shaped by the specific demands of the game and associated training methods. These sport-specific adaptations may influence both the execution of performance tests and the consistency of the results. In a recent study, the Isometric leg press and CMJ demonstrated high reliability when examining strength- and power-related variables in elite female ice hockey players [21], yet reliability data of a portable IMTP and CMJ in elite male ice hockey players are few. Although previous studies have not observed differences in CMJ reliability between untrained men and women [22,23], physiological differences, such as fiber composition and higher absolute forces and power outputs in males [24], may be more pronounced in elite athletes, potentially influencing the sensitivity and variability of CMJ and IMTP variables. Moreover, while the IMTP is a well-established tool for assessing maximal isometric strength, transitioning to a field-based portable set-up introduces new variables, including surface stability, anterior–posterior and medio-lateral movement of the bar, and environmental consistency, that may affect measurement reliability [9]. Hence, studies investigating the reliability of portable IMTP set-up and CMJ in elite male ice hockey players are warranted. This study aimed to establish the test–retest reliability of strength and power-related measures obtained from portable IMTP and CMJ measurements taken on different days in elite male ice hockey players.

## 2. Materials and Methods

### 2.1. Experimental Design

This study employed a test–retest reliability design to evaluate the measurement consistency of CMJ and portable IMTP assessments in elite male ice hockey players. The experimental protocol implemented a single-group repeated-measures design with participants completing identical assessments on two occasions, at the same time of the day, separated by 24 h. This interval minimized learning effects while preventing training-induced adaptations that could compromise reliability measurements [11]. All testing occurred during the off-season.

Each participant performed three maximal effort trials for both assessments during each session. Standardized conditions required participants to abstain from strenuous activity for 48 h before initial testing and avoid intensive lower-body training between sessions. Nutritional intake was replicated across testing days to minimize metabolic variables. Each session commenced with identical warm-up protocols consisting of 10 min of continuous cycling at 120 watts followed by 4 min of dynamic stretching and three IMTP warm-up trials at 50%, 75%, and 90% of maximal effort to ensure consistent physiological preparation across all testing procedures.

### 2.2. Participants

Twenty-two elite male ice hockey players from the Swedish Hockey League (SHL) participated in this study (age: 26.8 ± 5.1 years; height: 184.5 ± 3.9 cm; body mass: 88.5 ± 5.7 kg). All participants were injury-free and actively competing in the SHL, one of the world’s highest-ranked ice hockey leagues. Recruitment criteria required participants with a normal regular training volume of 8–10 training sessions per week. Participants also needed to include countermovement jump and maximal strength training for the lower limbs (>80% of 1RM) in their normal routine. Participants were excluded if they had interrupted or reduced training in the days preceding the first testing occasion due to injury or other health-related reasons, or if they had engaged in matches or intense training sessions within 48 h before testing. The sample size of 22 participants was determined based on a hypothetical power analysis, following the approach described by Borg et al. [25]. Since elite athletes typically demonstrate high measurement reliability, we anticipated low within-subject variation. Specifically, an intraclass correlation coefficient (ICC) above 0.90. Using this expected reliability and the commonly accepted threshold of ICC ≥ 0.75, we calculated the minimum sample size for adequate statistical power (α = 0.05, power = 0.80) as 12 participants. We included 22 participants to enhance the estimation precision and to reduce exclusion risk due to injury, non-compliance, or technical complications. All participants were fully informed about study procedures, risks, and benefits. They signed a health declaration and provided written informed consent before participation. The study was approved by the Regional Ethical Review Board in Stockholm, Sweden in 2025 (Dnr 2025-03038-01) and performed in accordance with the principles outlined in the Declaration of Helsinki.

### 2.3. Test Protocols

#### 2.3.1. Isometric Mid-Thigh Pull

**Figure 1:**
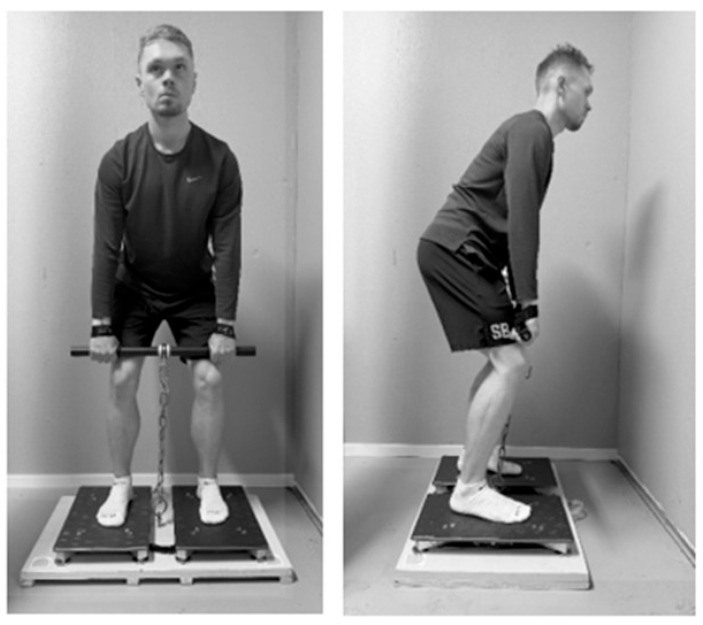
Representation of the portable isometric mid-thigh pull.

IMTP testing was performed on dual force plates (ForceDecks FD4000, Vald Performance, Brisbane, Australia) with participants in a standing position holding a bar attached to adjustable chains at mid-thigh level (hip angle ~145°, knee angle ~145°, see Figure 1). The force plates were zeroed before each trial to ensure accurate baseline measurements. Following bodyweight recording, participants applied ~100 N pretension before performing a 3 s maximal isometric pull initiated by a “3, 2, 1, GO” countdown. Wrist straps secured hands to the bar to eliminate grip strength effects. Body position was verified using a goniometer, and the participants were allowed a 3 min rest interval between each maximal trial. Force plate data were sampled at 1000 Hz and analyzed using Vald ForceDecks software (version 2.1.0). Peak force was recorded as the highest force value obtained during the trial. Rate of force development (RFD) was calculated as the maximal slope of the force-time curve (∆force/∆time) across multiple time windows: 0–50 ms, 0–100 ms, 0–150 ms, 0–200 ms, 0–250 ms, 50–100 ms, 100–150 ms, and 150–200 ms. Contraction onset was determined using a 20 N threshold above bodyweight. The highest average slope of the force–time curve within any 20 ms window, irrespective of contraction onset, was extracted as the RFD 20 ms moving average. Data were processed and analyzed using MATLAB (R2020a; The MathWorks, Natick, MA, USA). The best trial was used for final analysis.

#### 2.3.2. Countermovement Jump

CMJ testing was performed using the same force plate system (ForceDecks FD4000, Vald Performance, Brisbane, Australia) with identical software settings (1000 Hz sampling, 20 N movement detection threshold above bodyweight). Following two submaximal warm-up jumps with 60 s rest intervals, participants performed maximal CMJ trials with hands on hips (akimbo position). Standardized instructions included: (1) self-selecting countermovement depth, (2) remaining stationary for 3 s before jumping, (3) performing continuous movement without pausing, (4) jumping for maximum height, executing the concentric phase of the movement as fast as possible, and (5) landing centrally on the platform without lateral displacement. Variables analyzed from the best trial included jump height (impulse-momentum method), peak force, peak power, peak velocity, concentric and eccentric impulse, time to peak force, time to peak power, and concentric and eccentric duration.

### 2.4. Statistical Analyses

The normality of the data was checked with the Shapiro–Wilk test and heteroscedasticity testing was checked through Bland–Altman plots. Reliability was assessed using intraclass correlation coefficients (ICC_3_,_1_), coefficients of variation (CV), standard error of measurement (SEM), and minimal detectable change (MDC_95_) [26]. ICC interpretation followed established criteria: poor (<0.50), moderate (0.50–0.75), good (0.75–0.90), and excellent (>0.90) [27]. CV thresholds were set at ≤10% for acceptable reliability. Systematic bias was assessed using paired t-tests. All analyses were conducted using Jamovi 2.4.11 with significance set at *p* < 0.05.

## 3. Results

All measured parameters met the assumptions for parametric testing, with the Shapiro–Wilk test verifying normal distribution across all variables. Additionally, the data demonstrated homogeneity of variance (homoscedasticity), indicating that the variability in measurements was consistent across groups. here were no significant differences between test sessions for any analyzed variables (*p* > 0.05). Results from the countermovement jump (CMJ) and isometric mid-thigh pull (IMTP) assessments are summarized in Table 1 and Table 2, respectively.

IMTP Peak force demonstrated excellent reliability (ICC = 0.92) with low measurement error (CV = 3.1%). In contrast, rate-of-force parameters showed poor to moderate reliability, with ICC values ranging from 0.16 to 0.75. Only the 0–150 ms RFD variable achieved acceptable reliability (ICC = 0.75; 95% CI: 0.54–0.88), while most other RFD measures exhibited questionable reliability (ICC = 0.62–0.73). The 0–50 ms and 150–200 ms RFD intervals demonstrated particularly poor reliability (ICC = 0.57 and 0.16, respectively). Measurement precision varied considerably across RFD variables, with coefficient of variation values ranging from 12.9% (0–250 ms) to 54.6% (150–200 ms). Earlier time-windows (0–50 ms, 0–100 ms) exhibited exceptionally high measurement error (CV = 36.7–48.3%), indicating substantial random variability. Longer windows (0–150 ms, 0–200 ms) show better reliability than shorter windows (50 ms) or later windows (150–200 ms).

ICC ranged from good to excellent for all CMJ variables (0.76 to 0.96). The absolute CMJ variables (not adjusted to body mass) demonstrated excellent reliability (ICC = 0.93–0.96) while the CMJ variables adjusted for body mass ranged from good to excellent (ICC = 0.83–0.91). Peak power, peak velocity, and concentric and eccentric impulse variables demonstrated the most consistent measurement precision, with CV values ≤ 2.9%. For the CMJ variables, the highest CV values were observed for time to peak power, time to peak force, and eccentric duration (CV = 4.1–5.0%). CV values were generally lower for CMJ variables compared to the IMTP variables (CV = 1.4–5.0% compared to CV = 3.1–54.6%).

## 4. Discussion

This study evaluates the between-session reliability of a portable IMTP set-up and CMJ variables in elite male ice hockey players, providing novel insights into the limited body of research on the consistency of these commonly used performance measures in this specific population. The findings of this study demonstrate that countermovement jump and isometric mid-thigh pull measurements provide highly reliable measurements of explosive power and maximal strength in elite male ice hockey players. Good to excellent reliability was achieved for CMJ jump height, CMJ peak force, and IMTP peak force, confirming that commonly used strength and power variables can be measured with high reliability in male elite hockey players. This is consistent with previous findings in female players at the same competitive level [21].

Interestingly, concentric and eccentric impulses emerged as the most reliable variables across CMJ testing sessions, demonstrating superior consistency compared to peak force, jump height, and power. This finding is consistent with previous literature suggesting that impulse, as an integrated measure of force applied over time, is less susceptible to transient fluctuations and measurement noise typically observed in high-velocity movements [28]. Its robustness likely stems from its ability to capture the entire phase of the force-time curve, thereby providing a more stable and comprehensive representation of neuromuscular performance. Based on the high between-session reliability observed in our data, both concentric and eccentric impulse demonstrates strong potential as a diagnostic and monitoring tool in athletic populations, especially for tracking longitudinal performance changes and adaptations to training interventions. However, in sport-specific contexts where rapid force production within brief time frames is critical, impulse may lack ecological validity, as it prioritizes force-time integration over the immediacy of force expression. In addition, athletes may compensate for fatigue by modifying their technique, such as employing deeper countermovement phases to maintain impulse or jump height. The integrated nature of impulse may mask underlying fatigue effects or performance characteristics. Therefore, impulse may be most effectively utilized in combination with time-dependent variables, such as concentric phase duration, which showed excellent reliability, and may offer complementary insights into explosive performance characteristics.

Rate of force development demonstrated poor reliability in IMTP testing across all measured time intervals, with the notable exception of the 0–150 ms window, which exhibited moderate reliability. This pattern aligns with previous research, confirming that RFD calculations suffer from inherent measurement noise due to force-time differentiation, particularly problematic when assessing rapid force production in elite athletes [29]. The portable IMTP set-up likely introduced additional measurement variability compared to fixed laboratory setups. Rate of force development is highly sensitive to equipment stability and positioning consistency, methodological factors that likely contributed to the reduced reliability observed in RFD variables compared to peak force measurements [9]. Since the portable system (ForceDecks FD4000, Vald Performance, Brisbane, Australia) has demonstrated accuracy and reliability comparable to laboratory-grade fixed force platforms for both isometric and dynamic testing, the observed variability is unlikely to stem from the measurement equipment itself [30,31,32].

Previous research has indicated that IMTP force-time data accuracy may be influenced by contraction onset determination methods, with body weight-based approaches showing greater sensitivity [29,33]. However, the present analysis revealed no meaningful differences in reliability between methodological approaches. The moving mean method yielded an ICC of 0.68, remaining unaffected by onset thresholds, while fixed threshold methods with contraction onset defined as force exceeding 20 N above body weight produced comparable results (ICC range: 0.57–0.75).

In the present findings, outcome-oriented variables such as force, power, impulse, and jump height demonstrated greater reliability across countermovement jump (CMJ) testing sessions compared to time-dependent metrics like time to peak force, time to peak power, and phase durations. This suggests that variables reflecting overall performance outcomes are less susceptible to measurement noise and execution variability than those reliant on precise timing and phase identification. Moreover, absolute values consistently showed higher reliability than their body mass-normalized counterparts, likely reflecting the additional variability introduced during the normalization process. While time-dependent variables exhibited lower overall reliability, their reliability coefficients still ranged from good to excellent, indicating continued value in performance assessment protocols. However, caution is warranted when interpreting time to peak power, which demonstrated lower reliability and may be more vulnerable to inconsistencies in movement execution and signal processing. These findings support the use of outcome-oriented and absolute metrics as stable indicators of CMJ performance, while highlighting the need for careful consideration when incorporating time-sensitive variables into monitoring protocols.

Despite offering novel contributions, the present findings have limitations that should be acknowledged. The 24 h retest interval, while practically relevant, may not adequately capture longer-term measurement stability that is important for longitudinal monitoring. The sample size, although sufficient for reliability assessment, restricted the ability to conduct subgroup analyses by playing position or experience level. Examining reliability across different test intervals could help establish optimal monitoring frequencies for various applications, while position-specific analyses may reveal differences in reliability patterns linked to distinct physiological demands and training adaptations.

Although the participants were elite-level athletes with extensive training, the potential influence of internal factors, such as residual fatigue, motivation, and sleep quality, cannot be entirely excluded. Finally, integrating these assessments with on-ice performance measures would enhance the ecological validity of laboratory-based testing protocols.

### Practical Applications

Practitioners working with elite male ice hockey players can confidently implement CMJ and portable IMTP tests in their performance monitoring routines. CMJ variables such as jump height, peak force, and especially concentric and eccentric impulse are well-suited for longitudinal tracking due to their high test–retest reliability. Given the limitations of RFD in IMTP assessments, peak force should be prioritized when evaluating maximal strength, particularly in field-based environments. When profiling athletes, absolute values are generally more dependable than scaled metrics, especially in homogeneous populations like elite hockey players. The minimal detectable change thresholds identified in this study, such as 3.4 cm for jump height and 288 N for IMTP peak force, serve as objective benchmarks for distinguishing true performance adaptations from measurement variability in elite male ice hockey players. While normalization may be useful in certain contexts, its added complexity and reduced reliability warrant cautious use. Overall, combining robust variables with individualized baselines and objective thresholds enables practitioners to make informed decisions and tailor training interventions with greater confidence.

## 5. Conclusions

This study demonstrates that CMJ and portable IMTP can reliably assess key strength and power variables in elite male ice hockey players. CMJ impulse, peak velocity, peak force, and jump height, together with IMTP peak force, exhibited the highest consistency. In contrast, the IMTP rate of force development showed limited reliability, with only the 0–150 ms window demonstrating moderate consistency, emphasizing the need for caution when interpreting RFD. The present findings indicate that outcome-oriented and absolute variables should be prioritized, while selected time-sensitive variables may be incorporated to enhance diagnostic accuracy. These reliability assessments enable coaches and sport scientists to monitor ice hockey players with greater confidence, optimize training load management, refine recovery strategies, and support evidence-based return-to-play decisions.

## Figures and Tables

**Table 1:** Test–retest reliability of countermovement jump variables.

Variable	Session 1	Session 2	95% LoA	ICC (95% CI)	SEM	CV	MDC_95_
*Outcome-oriented variables*							
Peak jump height (cm)	41.9 ± 4.0	41.1 ± 3.9	0.8 (−2.8 to 4.4)	0.89 (0.79–0.95)	1.3	3.1	3.6
Peak force (N)	2244 ± 206	2240 ± 238	4 (−164 to 172)	0.93 (0.86–0.97)	59	2.7	164
Peak force·BM^−1^ (N·kg^−1^)	25.3 ± 1.6	25.2 ± 2.0	0.1 (−2.0 to 2.1)	0.83 (0.68–0.92)	0.7	2.9	2.0
Peak power (W)	4921 ± 472	4840 ± 435	81 (−229 to 391)	0.93 (0.88–0.97)	120	2.3	334
Peak power·BM^−1^ (W·kg^−1^)	55.6 ± 4.4	54.7 ± 4.4	0.9 (−2.6 to 4.4)	0.91 (0.83–0.96)	1.3	2.3	3.7
Peak velocity (m·s^−1^)	2.96 ± 0.14	2.94 ± 0.13	0.02 (−0.09 to 0.14)	0.94 (0.86–0.97)	0.03	1.4	0.09
Concentric impulse (N·s)	254 ± 20	251 ± 019	2.4 (−7.5 to 12)	0.96 (0.93–0.98)	3.9	1.4	10.7
Eccentric impulse (N·s)	138 ± 19	139 ± 18	−1.6 (−13 to 10)	0.95 (0.89–0.99)	4.1	2.9	11.3
Relative net impulse (N·s·kg^−1^)	5.63 ± 0.26	5.65 ± 0.25	−0.03 (−0.27 to 0.21)	0.88 (0.78–0.94)	0.09	1.5	0.24
*Time-dependent variables*							
Time to peak force (ms)	512 ± 57	508 ± 59	4 (−6 to 7)	0.86 (0.74–0.93)	022	4.5	60
Time to peak power (ms)	719 ± 66	719 ± 69	0 (−10 to 10)	0.76 (0.56–0.88)	033	5.0	90
Concentric duration (ms)	273 ± 23	275 ± 23	−4 (−22 to 13)	0.93 (0.85–0.96)	006	2.3	17
Eccentric duration (ms)	508 ± 47	503 ± 54	5 (−52 to 63)	0.83 (0.68–0.92)	021	4.1	58

Data are presented as mean ± standard deviation, n = 22. ICC = intraclass correlation coefficient (3,1); LoA = limits of agreement; SEM = standard error of measurement; CV = coefficient of variation (%); MDC_95_ = minimal detectable change at 95% confidence level.

**Table 2:** Test–retest reliability of isometric mid-thigh pull variables.

Variable	Session 1	Session 2	Bias (95% LoA)	ICC (95% CI)	SEM	CV	MDC_95_
Peak force (N)	3347 ± 388	3466 ± 344	−119 (−312 to 273)	0.92 (0.84–0.96)	104	3.1	288
*Rate of force development (N·s* ^−^^1^ *)*							
20 ms moving average	13,224 ± 6864	13,639 ± 4142	−414 (−10019 to 9190)	0.64 (0.37–0.81)	3402	25.8	9422
0–50 ms	4769 ± 4087	4430 ± 2310	339 (−5817 to 6495)	0.57 (0.26–0.77)	2178	48.3	6032
0–100 ms	6496 ± 4242	6978 ± 2812	−482 (−6672 to 8294)	0.62 (0.34–0.80)	2205	36.7	6108
0–150 ms	6602 ± 2639	6998 ± 2094	−396 (−3675 to 2882)	0.75 (0.54–0.88)	1211	17.8	3355
0–200 ms	6001 ± 1605	6447 ± 1605	−446 (−2745 to 1854)	0.73 (0.51–0.87)	837	13.4	2319
0–250 ms	5553 ± 1079	5839 ± 1451	−286 (−2331 to 1760)	0.67 (0.40–0.83)	734	12.9	2033
50–100 ms	8447 ± 4783	9832 ± 3938	−1385 (−8692 to 5921)	0.64 (0.36–0.81)	2595	28.2	7186
100–150 ms	8320 ± 2680	8917 ± 2813	−597 (−5067 to 3873)	0.66 (0.39–0.82)	1607	18.7	4452
150–200 ms	6328 ± 2668	6865 ± 2104	−537 (−6670 to 5996)	0.16 (−0.21–0.49)	2322	54.6	6432

Data are presented as mean ± standard deviation, n = 21. ICC = intraclass correlation coefficient (3,1); LoA = limits of agreement; SEM = standard error of measurement; CV = coefficient of variation (%); MDC_95_ = minimal detectable change at 95% confidence level.

## Data Availability

All data generated from this study can be found in the article.
